# Processing of Painful Pictures in Individuals With High and Low Rejection Sensitivity: Evidence From Event-Related Potentials

**DOI:** 10.1177/2041669519879722

**Published:** 2019-09-27

**Authors:** Wu Qinqin, Ran Guangming, Zhang Qi, Xiaojun Cao

**Affiliations:** Department of Psychology, Institute of Education, China West Normal University, Nanchong, China; College of Preschool and Primary Education, China West Normal University, Nanchong, China; Department of Psychology, Institute of Education, China West Normal University, Nanchong, China

**Keywords:** rejection sensitivity, vigilance, painful pictures, P100, late LPC

## Abstract

An increasing number of studies have investigated the relation between the processing of painful stimuli and rejection. Little was known, however, about the impact of the rejection sensitivity (RS) on the processing of painful pictures. This study addressed this issue using high temporal resolution event-related potential techniques. Thirty high RS (20 women and 10 men who scored in the top 20th percentile of the Rejection Sensitivity Questionnaire scores) and 30 low RS (20 women and 10 men who scored in the bottom 20th percentile) volunteers participated in the experiment. All volunteers performed a discrimination task of painful pictures in which they were asked to judge whether target pictures were painful or not. Behaviorally, participants exhibited shorter reaction times for painful than nonpainful pictures. For the P100 component, low RS participants showed stronger brain activities for painful than nonpainful pictures, suggesting vigilance toward painful pictures. High RS participants, however, exhibited no P100 amplitude differences between painful and nonpainful pictures, indicating an analgesia phenomenon. Furthermore, we found that there were larger amplitudes in the late late positive complex component for painful compared with nonpainful pictures, regardless of participants’ RS. This suggested a person’s further assessment for painful pictures. In short, our findings demonstrated that the level of RS influenced the pain processing at a very early stage of processing.

## Introduction

Rejection sensitivity (RS) is considered as the disposition to anxiously expect, readily perceive, and oversensitivity to rejection (Romero-Canyas, Downey, Berenson, Ayduk, & Kang, 2010). Some researchers have suggested that the RS influences persons’ think, feel, and behavior in their intimate relationships (Downey & Feldman, 1996). It is frequently associated with psychological difficulties and other adverse outcomes, such as relationship breakup, increasing depression, aggression, and mortality (Ayduk, Downey, & Kim, 2001; Kawamoto, Nittono, & Ura, 2015).

The threat value of pain hypothesis (TVPH) postulates that observing pain in others may be perceived as a threatening signal, leading to an activation of threat-detection system (Cui, Zhu, Duan, & Luo, 2015; Ibáñez et al., 2011; Meng et al., 2012). The TVPH suggests that low RS (LRS) individuals may show an automatic alarm response to painful pictures (a cortical discrimination between painful and neutral pictures) when they observe these stimuli (Cui et al., 2015; Ibáñez et al., 2011; Meng et al., 2012). However, high RS (HRS) ones, who have experienced severe forms of social exclusion, would tend to avoid/escape threat stimuli (an inhibition processing for threatening signal), leading to an analgesia phenomenon when they encountered painful pictures (Bernstein & Claypool, 2012; Dewall & Baumeister, 2006; Macdonald & Shaw, 2005). In sum, the TVPH suggests that RS and pain processing are related. More specifically, the level of RS influences a person’ pain processing. Our study is the first to adopt high temporal resolution event-related potential (ERP) techniques to explore the impact of the RS on the pain-cue processing (the processing of painful pictures).

A large number of ERP studies have explored the processing of painful pictures. The P100, an early ERP component, is commonly thought to reflect early visual processing (Ran & Chen, 2017; Ran, Zhang, Chen, & Pan, 2014; Zhang, Ran, & Li, 2018). An ERP study examining the processing of pain in others has shown that one early allocation of attention as reﬂected by higher P100 amplitudes is related to viewing more rather than less arousing images (Lyu, Meng, & Jackson, 2014), suggesting that painful pictures may evoke higher P100 amplitudes than neutral ones as painful stimuli are high-arousing images. In addition, larger P100 amplitudes have been found in the processing of cues of negative emotion and threat (Ran, 2018; Ran & Chen, 2017; Sass et al., 2010). Although some reports do not detect effects of pain on P100 amplitudes (Meng et al., 2012, 2013), several other studies observe lower P100 amplitudes for painful than nonpainful pictures (Dittmar, Baum, Schneider, & Lautenbacher, 2015; Figure 3 in Lautenbacher et al., 2013). One can speculate that these inconsistencies may be due to differences in experimental stimuli. The picture stimuli showing accidents in everyday life have been adopted in the studies conducted by Meng et al. (2012, 2013). However, facial pictures have been employed in the study of Dittmar et al. (2015).

Following the P100, the early posterior negativity (EPN) component is believed to result from automatic selective visual attention toward emotional stimuli (Schupp, Junghofer, Weike, & Hamm, 2004; Van Strien, Franken, & Huijding, 2014). There is evidence that individuals show larger EPN amplitudes for painful than neutral pictures in a visual discrimination task (Dittmar et al., 2015; Fabi & Leuthold, 2018) and latter EPN peak latencies for painful than angry faces in a recognition task of dynamic expressions (Ptito, Missana, Grigutsch, & Grossmann, 2014). A late ERP positive component is the P300, which reflects the arousing content of stimuli (Keil et al., 2002). The pain effect in the P300 (larger P300 amplitudes for painful stimuli) has been observed in the study of Han, Fan, and Mao (2008). Another ERP late positive deflection, the late positive complex (LPC), has been examined in a number of studies of painful picture processing (Cui, Ma, & Luo, 2016; Meng et al., 2013; Wang, Ge, Zhang, Liu, & Luo, 2014; Weng, 2010). For example, a recent ERP study has found that painful pictures elicit larger LPC amplitudes than nonpainful pictures (Cui et al., 2016).

Some studies have found that adolescents and adults respond more quickly to painful than nonpainful pictures in a discrimination task of painful pictures (Li & Han, 2010; Mella, Studer, Gilet, & Labouvie-Vief, 2012). The TVPH posits that both the early and the late processing of painful stimuli are associated with a potential threat (Cui, Zhu, & Luo, 2017; Ibáñez et al., 2011). Therefore, we expected a pain effect at the behavioral level and electrophysiological level (in the P100, EPN, P300, and LPC component). Given that the TVPH implies that LRS individuals should show an automatic alarm response to painful pictures while HRS ones would demonstrate an analgesia phenomenon, we hypothesized that painful pictures would evoke enhanced ERP amplitudes in the LRS but not in the HRS individuals.

## Materials and Methods

### Participants

Sixty volunteers (40 women and 20 men; mean age = 20.38 years, standard deviation [*SD*] = 2.02 years; all right-handed) with no history of neurological, psychiatric, or visual impairments were preselected from a group of 532 undergraduate students based on their RS scores in the Chinese version of the Rejection Sensitivity Questionnaire (RSQ) translated by Zhao, Li, and Zhang (2012). The study of Zhao et al. (2012) found that the Chinese RSQ was a reliable and valid measure (e.g., Cronbach’s α coefficient = .835; test–retest reliability coefficient = .850). On the basis of previous studies (Ehrlich, Gerson, Vanderwert, Cannon, & Fox, 2015; Kraines, Kelberer, & Wells, 2018; Kross, Egner, Ochsner, Hirsch, & Downey, 2007), HRS participants (*N* = 30, 20 women) were defined as those who scored in the top 20th percentile of the RSQ scores, while the LRS participants (*N* = 30, 20 women) were those scoring in the bottom 20th percentile. There was a significant difference in the RSQ scores between HRS and LRS participants, HRS: 11.97 ± 4.98, LRS: 4.89 ± 2.37; *t*(58) = −7.14, *p* < .001. No group difference, however, was found for participants’ age, HRS: 20.67 ± 2.47, LRS: 20.10 ± 1.42; *t*(58) = −1.09, *p* = .28 (Table 1). All participants were provided written informed consent and received course credit for their participation. The study was approved by the local ethics committee, and the experiments were carried out in accordance with the approved guidelines.

**Table 1.** Participants’ Characteristics for HRS and LRS Participants.

	HRS participants (*N* = 30)	LRS participants (*N* = 30)
RSQ score	11.97 (4.89)	4.89 (2.37)
Age	20.67 (2.47)	20.10 (1.42)

*Note.* RSQ score = Rejection Sensitivity Questionnaire score; HRS = high rejection sensitivity; LRS = low rejection sensitivity.

### Materials

Target pictures consisted of 80 digital color pictures (40 painful and 40 nonpainful pictures) showing people’s hands, forearms, or feet (Meng et al., 2012, 2013). All pictures showed accidents that occurred in everyday life, such as a hand trapped in a door, a hand cut by a knife, or a foot touched by a pencil (Meng et al., 2012). Each picture had dimensions of 8.99 × 6.76 cm (width × height) and 100 pixels per inch. Meng et al. (2012) explicitly guaranteed that luminance, contrast, and color were matched between the painful and nonpainful pictures. On the basis of the study of Meng et al. (2012), the painful and nonpainful pictures were assessed by using 9-point Likert-type scales. There was a significant difference in the pain intensity between the painful and nonpainful pictures, painful pictures: 5.93 ± 0.71; nonpainful pictures: 2.13 ± 0.47; *t*(78) = −28.06, *p* < .001. The viewing angle of each image was 2.8 × 3.7°, with a screen resolution of 72 pixels per inch. The serial order of the pictures (painful vs. nonpainful pictures) was randomized across the experimental trials.

### Procedure

**Figure 1.**
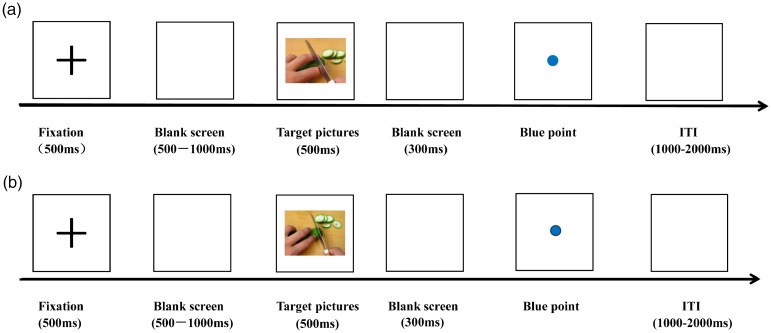
Schematic illustration of the experimental procedure (a) painful picture trials; (b) nonpainful picture trials). ITI = intertrial interval.

Participants seated comfortably 90 cm from the computer screen and were instructed to try their best to avoid head movements and eye blinks. While their electroencephalography (EEG) data were acquired, participants performed a discrimination task of painful pictures in which they were asked to judge whether target pictures were painful or not (Fan & Han, 2008; Li & Han, 2010). The task consisted of two blocks of 60 trials, yielding a total of 120 trials per participant. Each trial of the experiment started with a black cross (“+”), which was concentrated on a white background for 500 milliseconds (Figure 1). After a blank screen was displayed for a randomized amount of time (500–1,000 milliseconds), a target picture (a painful or nonpainful picture) was presented for 500 milliseconds. Following the target stimulus, one blank screen was depicted for 300 milliseconds and subsequently a blue point was presented at the center of a computer screen. The intertrial interval was 1,000–2,000 milliseconds. Upon observing the blue point, the participants were asked to perform the discrimination task of painful pictures. Half of the participants were instructed to respond as quickly and accurately as possible by pressing the “1” key on the keyboard every time when they observed a painful picture and the “2” key for a nonpainful picture, whereas the other half of the participants were asked to use a reversed key arrangement. Four practice trials were given prior to starting the main trials.

### EEG Recording

The EEG was recorded at 64 scalp sites using tin electrodes mounted in an elastic cap (Brain Products, Munchen, Germany), with a reference on FCz electrode (Haueisen, Fiedler, Zanow, & Fonseca, 2017; Ran & Chen, 2017; Wu, Gu, & Zhang, 2015). The vertical electrooculogram (EOG) was recorded with an electrode placed below the right eye and the horizontal EOG was recorded from the right orbital rim. The interelectrode impedance was maintained below 5 kΩ. The EEG and EOG activities were amplified using a 0.01 to 100 Hz bandpass and continuously sampled at 500 Hz per channel.

### Behavioral and EEG Analysis

For behavioral analysis, a repeated-measure analyses of variance (ANOVAs), with picture stimuli (painful vs. nonpainful pictures) as a within-participant factor and group (HRS vs. LRS participants) as a between-participant factor, was performed on participants’ reaction times (RTs) and accuracies. For ERP analysis, EEG data in each condition were aligned and averaged separately. The EEG data were recomputed to average mastoid reference (Chen, Ran, Zhang, & Hu, 2015; Ran & Chen, 2017). They were further filtered off-line (0.05–30 Hz bandwidth). Ocular artifacts (blinks and other movements) were corrected using a Gratton and Coles-based algorithm off-line. Trials with artifacts exceeding 100 µV mainly due to amplifier clippings and peak-to-peak deflections were omitted from the averaging. The EEG data were segmented from −200 to 1,000 milliseconds relative to the target pictures, with a 200 milliseconds prestimulus baseline.

According to the study of Ran and Chen (2017), the P100 component was determined over O1/PO3/P3 (left hemisphere) and O2/PO4/P4 (right hemisphere) electrodes. Time window of the P100 component was defined as the most positive peak at 70 to 130 milliseconds (Ran & Chen, 2017). Following the P100, the EPN was scored as the mean activity at occipital electrodes (left hemisphere: PO3/O1, midline hemisphere: POz/Oz, and right hemisphere: PO4/O2) in the 225 to 300 milliseconds time window after picture onset (Van Strien et al., 2014). In addition, the P300 component was analyzed within a time frame of 350 to 450 milliseconds after stimulus onset at FC3/C3/CP3/P3 (left hemisphere), FCz/Cz/CPz/Pz (midline hemisphere), and FC4/C4/CP4/P4 (right hemisphere) electrodes (Meng et al., 2012). As for the LPC component, on the basis of previous literature (Dittmar et al., 2015; Lautenbacher et al., 2013), we distinguished the LPC between an early activation at 260 to 460 milliseconds (early LPC) and a late activation at 460 to 800 milliseconds (late LPC). The early and late LPC components were measured at the following sites: left (C3/P3), midline (Cz/Pz), and right hemisphere (C4/P4; Cui et al., 2015; Meng et al., 2012). Mean amplitudes of these components were subjected to repeated-measures ANOVA with picture stimuli and hemisphere as within-participant factors and group as a between-participant factor. The ERP data were analyzed off-line with Brain Vision Analyzer (Brain Products; Gilching, Germany). All degrees of freedom for the *F* ratio were corrected according to the Greenhouse–Geisser method.

## Results

### Behavioral Results

The data of RTs and mean accuracies for each condition were displayed in Table 2. Analysis of the RT data yielded a main effect of picture stimuli, *F*(1, 58) = 14.39, *p* < .001, ${\text{η}}_{\text{p}}^{\text{2}}$ = .20, responding more quickly for painful (*M* = 411.82 milliseconds, *SD* = 142.55 milliseconds) than nonpainful pictures (*M* = 483.40 milliseconds, *SD* = 208.02 milliseconds). However, the main effect of group, *F*(1, 58) = 0.001, *p* = .971, ${\text{η}}_{\text{p}}^{\text{2}}$ < .001, and the interaction between picture stimuli and group, *F*(1, 58) = 0.35, *p* = .558, ${\text{η}}_{\text{p}}^{\text{2}}$ = .01, failed to reach significance. With regard to mean accuracy data, no significant effects were found (all *F*s < 0.86, *p*s > .357).

**Table 2.** Means and *SD*s of RTs and Accuracies for HRS and LRS Group in Painful and Nonpainful Picture Trials.

Picture stimuli	RTs (ms)				Accuracies (%)			
	HRS		LRS		HRS		LRS	
	*M*	*SD*	*M*	*SD*	*M*	*SD*	*M*	*SD*
Painful	418.14	158.90	405.50	126.53	94.49	3.67	93.66	4.82
Nonpainful	478.60	236.91	487.19	178.49	94.79	3.46	94.05	4.22

*Note.* RT = reaction time; *SD* = standard deviation; HRS = high rejection sensitivity; LRS = low rejection sensitivity.

### ERP Results

#### P100 (70–130 milliseconds)

**Figure 2.**
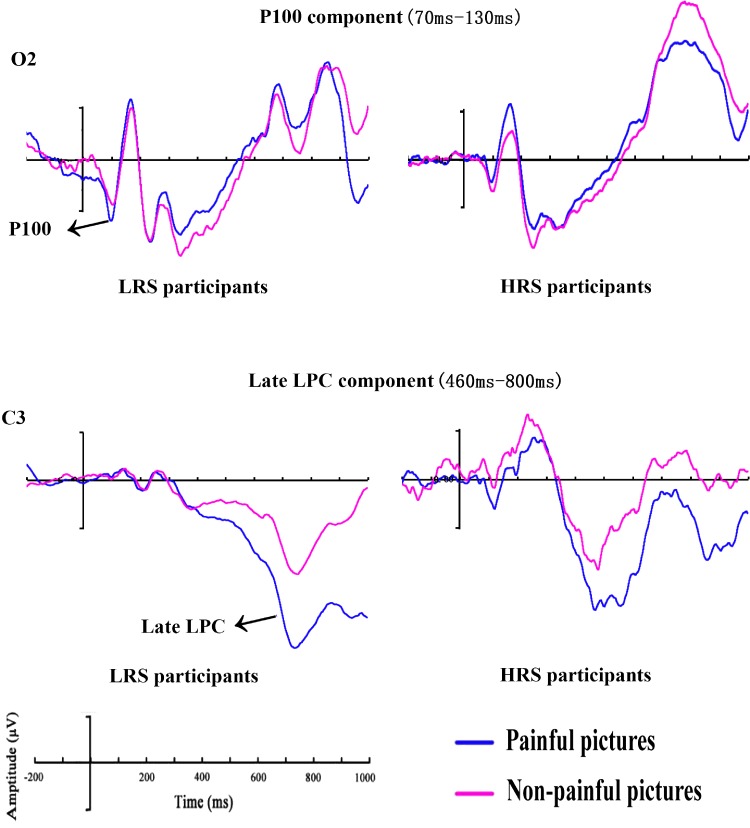
Grand mean ERPs between HRS and LRS individuals for painful and nonpainful picture trials at O2 electrode with time windows of the P100 and C3 electrode with time windows of the late LPC. HRS = high rejection sensitivity; LPC = late positive complex; LRS = low rejection sensitivity.

The ANOVA of P100 mean amplitudes showed a significant main effect of picture stimuli, *F*(1, 58) = 17.87, *p* < .001, ${\text{η}}_{\text{p}}^{\text{2}}$ = .24. The main effect for hemisphere was also significant, *F*(1, 58) = 6.66, *p* = .012, ${\text{η}}_{\text{p}}^{\text{2}}$ = .10. Furthermore, there was a significant interaction between group and picture stimuli, *F*(1, 58) = 4.21, *p* = .045, ${\text{η}}_{\text{p}}^{\text{2}}$ = .07. Follow-up analyses showed that painful pictures evoked a larger response than nonpainful pictures for LRS participants (painful pictures: *M* = 5.55 µV, *SD* = 6.85 µV; nonpainful pictures: *M* = −1.27 µV, *SD* = 5.99 µV; *p* < .001) but not for HRS participants (painful pictures: *M* = 3.57 µV, *SD* = 4.89 µV; nonpainful pictures: *M* = 1.21 µV, *SD* = 4.94 µV; *p* = .130; Figure 2). The interaction between hemisphere and picture stimuli was also significant, *F*(1, 58) = 9.04, *p* = .004, ${\text{η}}_{\text{p}}^{\text{2}}$ = .14. Further analyses revealed more positive amplitudes in the right than left hemisphere electrodes for the nonpainful picture trials (right hemisphere: *M* = 1.82 µV, *SD* = 5.39 µV; left hemisphere: *M* = −1.89 µV, *SD* = 8.07 µV; *p* = .001) but not for the painful picture trials (right hemisphere: *M* = 4.56 µV, *SD* = 6.08 µV; left hemisphere: *M* = 4.57 µV, *SD* = 7.54 µV; *p* = .987). No other significant amplitude differences were observed in this component (all *F*s < 1.95, *p*s > .168).

#### EPN (225–300 milliseconds) and P300 (350–450 milliseconds)

The analysis of EPN component yielded no significant main effects, *picture stimuli: F*(1, 58) = 0.10, *p* = .754, ${\text{η}}_{\text{p}}^{\text{2}}$ = .002; *hemisphere*: *F*(2, 116) = 0.48, *p* = .496, ${\text{η}}_{\text{p}}^{\text{2}}$ = .01; *group*: *F*(1, 58) = 2.19, *p* = .145, ${\text{η}}_{\text{p}}^{\text{2}}$ = .04, or interaction effects, Picture Stimuli *×* Group: *F*(1, 58) = 0.43, *p* = .513, ${\text{η}}_{\text{p}}^{\text{2}}$ = .01; Picture Stimuli × Group × Hemisphere: *F*(2, 116) = 1.81, *p* = .184, ${\text{η}}_{\text{p}}^{\text{2}}$ = .03. No other significant amplitude differences were observed on this component (all *F*s < 0.76, *p*s > .390). In addition, no significant effects were found in P300 component (all *F*s < 2.33, *p*s > .113).

#### Early (260–460 milliseconds) and Late (460–800 milliseconds) LPC

**Figure 3.**
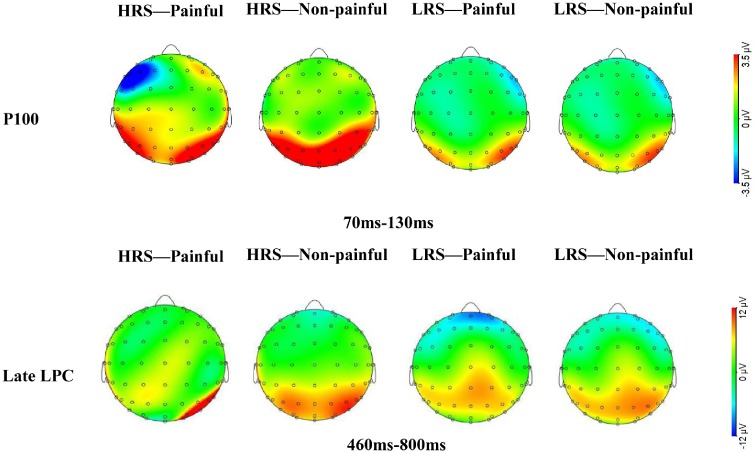
HRS and LRS individuals’ scalp topographies of ERP generated by painful and nonpainful pictures at the P100 and late LPC components. HRS = high rejection sensitivity; LPC = late positive complex; LRS = low rejection sensitivity.

In the analysis of the early LPC mean amplitudes, none of the main effects or interactions reached significant (all *F*s < 1.59, *p*s > .212). The ANOVA for the late LPC mean amplitudes yielded a significant main effect of picture stimuli, *F*(1, 58) = 8.35, *p* = .005, ${\text{η}}_{\text{p}}^{\text{2}}$ = .13 (Figure 2). Follow-up analyses confirmed that there were larger amplitudes (more positive amplitudes) for painful (*M* = 6.96 µV, *SD* = 20.52 µV) than nonpainful pictures (*M* = −3.66 µV, *SD* = 26.97 µV). No other main effects and interactions reached significance, *hemisphere*: *F*(2, 116) = 1.50, *p* = .277, ${\text{η}}_{\text{p}}^{\text{2}}$ = .03; *group*: *F*(1, 58) = 0.96, *p* = .333, ${\text{η}}_{\text{p}}^{\text{2}}$ = .02; Picture Stimuli × Group: *F*(1, 58) = 0.81, *p* = .371, ${\text{η}}_{\text{p}}^{\text{2}}$ = .01; Picture Stimuli × Hemisphere: *F*(2, 116) = 0.40, *p* = .597, ${\text{η}}_{\text{p}}^{\text{2}}$ = .01; Group × Hemisphere: *F*(2, 116) = 0.39, *p* = .623, ${\text{η}}_{\text{p}}^{\text{2}}$ = .01; Picture Stimuli × Group × Hemisphere: *F*(2, 116) = 0.341, *p* = .634, ${\text{η}}_{\text{p}}^{\text{2}}$ = .01. The grand average ERP topographies of the P100 and late LPC components in each condition were shown in Figure 3.

## Discussion

This study employed the discrimination task of painful pictures to examine the processing of painful pictures in individuals with HRS and LRS. Behaviorally, we found that participants displayed shorter RTs for painful compared with nonpainful pictures. For the P100 component, LRS participants showed notably enhanced amplitudes in response to painful compared with nonpainful pictures. However, no increased P100 amplitudes for painful compared with nonpainful pictures were observed in HRS participants. Furthermore, greater late LPC amplitudes were detected in the painful picture trails, regardless of the participants’ RS.

This work revealed a pain effect at the behavioral level, evidenced by participants’ shorter RTs to painful pictures. This was consistent with the finding of a previous study (Li & Han, 2010). It was noteworthy that Li and Han (2010) adopted the same task (the discrimination task of painful pictures) and applied the similar painful pictures as ours. A recent study, however, reported that participants’ RTs were significantly longer for painful pictures than nonpainful pictures in a prime-target paradigm (Meng et al., 2012). In sum, these results revealed that the pain effect was influenced by the tasks used in the studies.

It has previously been argued that enhanced P100 amplitudes were associated with early visual attention (Alonso et al., 2015; Kappenman, Farrens, Luck, & Proudfit, 2014; Ran et al., 2014; Spironelli & Angrilli, 2011). Although some previous studies reported lower P100 amplitudes to painful than neutral pictures (Dittmar et al., 2015; Figure 3 in Lautenbacher et al., 2013) or detected no effects of pain on P100 amplitudes (Meng et al., 2012, 2013), this study observed that LRS participants exhibited increased P100 amplitudes when they perceived painful pictures, presumably reflecting an early vigilance to painful pictures. There was evidence that painful stimuli were biologically important for human beings (Babiloni et al., 2004), resulting in the early allocation of attention to these stimuli, and ultimately increasing LRS participants’ vigilance to them.

Interestingly, HRS participants showed no P100 amplitude differences between painful and nonpainful pictures. The lack of differentiated processing of the pictures (painful vs. nonpainful pictures) in HRS participants was compatible with an argument that HRS participants showed an analgesia phenomenon as they were severe oversensitivity to rejection (Bernstein & Claypool, 2012; Dewall & Baumeister, 2006; Macdonald & Shaw, 2005). Some researchers have suggested that high level of social exclusion experience may disrupt the ability to respond to physical pain, leading to increases in both pain threshold (e.g., sensitivity to pain) and pain tolerance (e.g., withstanding greater pain; Bernstein & Claypool, 2012; DeWall & Baumeister, 2006). Such analgesia to painful stimuli was also observed in the patients with affective illness (Davis, Buchsbaum, & Bunney, 1979). The RS-group differences were observed in P100, but not in RT data. This might be due to the fact that the P100 was so early that it would not pick up such processes in RTs. It was noteworthy that the moderating effect of the level of RS on the pain processing was found in the P100 but not EPN, P300 and LPC component. This result suggested that the level of RS influenced the pain processing at a very early stage of processing.

This study observed larger late LPC amplitudes for painful compared with nonpainful pictures, regardless of participants’ RS. This result was in line with several empirical findings that found a pain effect in the late LPC (Cui et al., 2016; Meng et al., 2013; Weng, 2010). The late LPC pain effect indicated a person’s further assessment for painful pictures because the late LPC component was generally associated with a top-down cognitive assessment processing of visual stimuli (Cheng, Jiao, Luo, & Cui, 2017; Wang et al., 2014). We failed to observe the pain effects in the EPN, P300, and early LPC component, which was inconsistent with our hypotheses. Such inconsistency should be examined in future studies.

This study provides the first demonstration of how individual differences in RS affect the processing of painful pictures. Moreover, our findings extend previous RS research by showing the modulation of early but not late ERPs by the RS. Like other studies, this study is not without limitations. For example, our study is only comparing two picture categories (pain and nonpain). Hence, our findings cannot be attributed specifically to the pain-related content of the pictures. It may be pain that matters or it is only threat in general or something of negative valence.

## Conclusion

Although a wealth of research has examined the RS (e.g., Ehrlich et al., 2015; Park & Lee, 2018), there was no study that directly investigated the processing of painful pictures in individuals with HRS and LRS using the ERP techniques. This study reported a pain effect at the behavioral level. The ERP results from the P100 component revealed larger amplitudes for painful than nonpainful pictures among LRS participants, suggesting that they showed vigilance to painful pictures. HRS participants, however, exhibited no larger P100 amplitudes for painful than nonpainful pictures, indicating an analgesia phenomenon. As for the late LPC, there were larger amplitudes for painful compared with nonpainful pictures regardless of participants’ RS, which implied a person’s further assessment for painful pictures.
